# Clinical Perspectives on Cochlear Implantation in Pediatric Patients with Cochlear Nerve Aplasia or Hypoplasia

**DOI:** 10.3390/audiolres15040096

**Published:** 2025-08-05

**Authors:** Ava Raynor, Sara Perez, Megan Worthington, Valeriy Shafiro

**Affiliations:** 1 Children’s Hospital of Philadelphia, 3401 Civic Center Blvd, Philadelphia, PA 19104, USA; raynora@chop.edu; 2 Communication Disorders and Sciences, Rush University, 600 S. Paulina Street, Chicago, IL 60612, USA; sara_i_perez@rush.edu (S.P.); megan_m_worthington@rush.edu (M.W.)

**Keywords:** cochlear nerve deficiency, cochlear nerve aplasia, cochlear near hypoplasia, cochlear implant, auditory brainstem implant, clinical decision making

## Abstract

**Background**: Cochlear implantation (CI) in pediatric patients with cochlear nerve deficiencies (CND) remains controversial due to a highly variable clinical population, lack of evidence-based guidelines, and mixed research findings. This study assessed current clinical perspectives and practices regarding CI candidacy in children with CND among hearing healthcare professionals in the USA. **Methods**: An anonymous 19-question online survey was distributed to CI clinicians nationwide. The survey assessed professional background, experience with aplasia and hypoplasia, and perspectives on CI versus auditory brainstem implant (ABI) candidacy, including imaging practices and outcome expectations. Both multiple-choice and open-ended responses were analyzed to identify trends and reasoning. **Results**: Seventy-two responses were analyzed. Most clinicians supported CI for hypoplasia (60.2%) and, to a lesser extent, for aplasia (41.7%), with audiologists more likely than neurotologists to favor CI. Respondents cited lower risk, accessibility, and the potential for benefit as reasons to attempt CI before ABI. However, many emphasized a case-by-case approach, incorporating imaging, electrophysiological testing, and family counseling. Only 22.2% considered structural factors the best predictors of CI success. **Conclusions**: Overall, hearing health professionals in the USA tend to favor CI as a first-line option, while acknowledging the limitations of current diagnostic tools and the importance of individualized, multidisciplinary decision-making in CI candidacy for children with CND. Findings reveal a high variability in clinical perspectives on CI implantation for pediatric aplasia and hypoplasia and a lack of clinical consensus, highlighting the need for more standardized assessment and imaging protocols to provide greater consistency across centers and enable the development of evidence-based guidelines.

## 1. Introduction

Cochlear implants (CI) have provided an effective intervention for children with severe-to-profound hearing loss, improving quality of life and economic outcomes [1]. As a neuroprosthetic device, a CI bypasses damaged inner ear structures and stimulates the cochlear nerve (CN) directly through the application of the electrical current [2]. Thus, effective CI functioning is contingent on the sufficient integrity of the central auditory nervous system, starting with the CN. If the CN is compromised and the number of afferent spiral ganglion neurons is too low, the path for electrical stimulation from the CI to central brain regions will be impeded, leading to poor treatment outcomes. Therefore, CN deficiencies (CND), such as aplasia and hypoplasia, have historically been considered contraindications for receiving a CI [3], with an Auditory Brainstem Implant (ABI) as a potential alternative [3,4,5,6]. However, ABI may also pose an increased risk [3,7] and result in more limited auditory outcomes due to its more central placement. Extant research generally indicates mixed findings about both ABI and CI outcomes with CND [4,8,9,10]. Given the lack of consensus, large patient variability, and a limited evidence base, it is important to examine the state of current clinical practices and recommendations about implantation in cases of pediatric CND. The current project investigated clinical perspectives on CI in children with CN aplasia and hypoplasia in relationship to clinical decision-making. The findings were expected to highlight the current clinical perspectives and practices and provide a basis for the future development and implementation of evidence-based clinical guidelines to improve patient outcomes for children with CND.

Past research has indicated that 25–48% of patients with a congenital unilateral hearing loss and 15.4% of patients with a severe-to-profound bilateral hearing loss are associated with CND [11]. The diagnosis of CND relies on using imaging and electrophysiological studies to assess the functional and anatomical characteristics of the nerve, following audiologic assessment. Current best practices for diagnosis of CN aplasia or hypoplasia involve high-resolution T2 sequence magnetic resonance imaging (MRI) [3,4,10,12,13,14]. In addition, high-resolution computed tomography (HRCT) can also be used to visualize bony labyrinth anomalies and inform surgical planning [12,15]. However, imaging results may be inconclusive, thus introducing a large degree of uncertainty about potential CI outcomes.

To provide a stronger and more systematic guidance for achieving the success of CI based on MRI, Govaerts et al. [16] created a grading scale for structural deficits of inner ear anatomy based on the affected branch of the nerve and degree of labyrinthine dysplasia. In a later study, Birman et al. [17] developed an expanded grading criterion, citing the need for improvement due to increased MRI resolution. However, Kari et al. [14] found limited success with the Birman CN classification. Several other studies similarly indicate that pre-operative imaging currently cannot reliably predict CI outcomes and potential benefit [10,13,18]. This may be due to the current resolution in MRI resulting in incomplete visualization [3,4,7,10,14,19], a lack of consistent protocols during imaging and in interpretation [14], and disagreement about what constitutes hypoplasia [3].

In addition to imaging, electrically evoked compound action potentials (ECAPs) [13,19] and elicited cortical auditory evoked potentials (eCAEPs) [19] have also been used to assess the functional integrity of the central auditory nervous system as potential predictors of CI success. He et al. [19] compared eCAEP responses of children with CI with and without CND. Their results showed that while an absent eCAEP was indicative of poor performance, present or robust eCAEP responses were not necessarily indicative of high performance on behavioral outcomes, as assessed by the Phonetically Balanced Kindergarten (PBK) test and behavioral audiometry. ECAP responses did not consistently match eCAEP responses in this study, and were also not predictive of outcomes on these behavioral tests, with issues of potential confounding of information from neural generators within the vestibular system. Similarly, Chao et al. [13] examined the relationship between ECAP responses and radiological findings in children with CIs diagnosed with aplasia but no other apparent development delays or genetic hearing loss syndromes. Specifically, the authors examined associations of ECAP responses with the width of the bony cochlear nerve canal (BCNC) and with the diameter of the vestibulocochlear nerve (VCN). Their results did not find a significant correlation between absent or positive ECAP responses and BCNC or VCN imaging findings.

Given the mixed research findings and the lack of consensus in published literature on the subject, it is not surprising that no implantation guidelines specific to pediatric patients with CND have been developed. The American Cochlear Implant Alliance’s most recent general guidelines for CI candidacy in pediatric populations do not address CND directly, but state that candidacy determination is highly variable and individualized to CI centers [20]. Consideration of several factors is emphasized when determining candidacy: (1) demographic characteristics and lifestyle, (2) audiologic evaluation, (3) medical evaluation/status, (4) speech and language evaluation, (5) counseling and therapy, and (6) other considerations, including comorbidities and insurance coverage. While the guidelines do not directly address aplasia or hypoplasia, it is noted that children with atypical cochlear anatomy have poorer postoperative outcomes, with narrow internal auditory canals and common cavity malformations being the most indicative. This aligns with Freeman & Sennaroglu’s recommendations [3], which cautions that CI outcomes are generally poorer in cases of aplasia or hypoplasia than those with typical cochlear nerves, with Categorical Auditory Performance (CAP) scores of less than five (a CAP score of 5 indicates the ability to understand common phrases without lipreading).

In the absence of clear guidelines, professional opinions about treatment plans with CI utilization in children with CND may vary widely, leading to inconsistent practices, which may in turn result in undesirable outcomes. It is important to understand how decisions about CI candidacy in pediatric CND cases are being made and identify potential discrepancies. To that end, the current study examined perspectives and practices of hearing healthcare providers in the United States regarding CI and ABI for children with CND to elucidate preferences and rationales for choosing an appropriate treatment approach.

## 2. Materials and Methods

To investigate clinical perspectives on implantation in children with CNDs, aplasia, and hypoplasia, we conducted an anonymous electronic survey using REDCap electronic data capture tools [21]. Following the approval of the Rush University Institutional Review Board (23012201-IRB01), the survey containing 19 questions was distributed to academic medical centers and clinics in the United States of America with active CI programs and posted in online professional forums. The clinical sites were identified through the American Cochlear Implant Alliance contact list and the professional networks of the investigators. Contacts at clinical sites were encouraged to share the survey with other professionals with CI experience. Although this convenience sampling method may result in selection bias, overrepresenting clinicians with strong opinions or greater confidence, it was chosen to elicit initial input from a specialized group of hearing health care professionals.

Nineteen survey questions (Appendix A) were developed based on key issues discussed in extant clinical literature on the topic of CI with CND [10,16,17] and were informed by discussions with individual clinicians with experience in this area. The survey questions assessed participants’ background—current professional role, CI experience, years of practice, and examined their perspectives on implantation recommendations with CNDs, as well as reasoning behind their choices. The survey included questions aimed at covering a broad range of relevant issues, including candidacy criteria, protocols, outcome measures, and the use of imaging.

Of the 19 questions, 11 were multiple-choice and 8 were open-ended questions. The first three questions assessed participants’ professional background (setting, role, and CI experience). To ensure maximum inclusivity of perspectives, all survey responses were collected anonymously, and no questions were mandatory. Open-ended questions were designed to give participants an opportunity to explain their reasoning for responses given and allow for more individualized responses.

Collected survey responses were characterized based on their completeness and participants’ professional roles and experience. Responses that did not indicate their professional background were excluded. Categorical responses to multiple choice questions were characterized by percentages of options selected. These results were subsequently used to guide an analysis of free-text responses to identify common themes in reasoning that support specific opinions and treatment preferences. When questions were skipped, partial responses were retained when they provided relevant data to specific questions. This decision was based on the exploratory design of the study, which aimed to capture a broad range of clinical perspectives.

## 3. Results

A total of 76 survey responses were collected. Four participants did not provide any information about their professional background, and their responses were excluded from further analysis. Of the remaining 72 responses, 42 were fully completed and 30 partially completed. Participants’ characteristics are shown in Table 1. Most of the 72 participants whose responses were analyzed had six or more years of CI experience (81%), identified as audiologists (53%) and either neurotologists (40%) or otolaryngologists (4%), worked at a clinical site with a CI program (96%), and specifically worked with children with aplasia/hypoplasia (50%).

**Figure 1:**
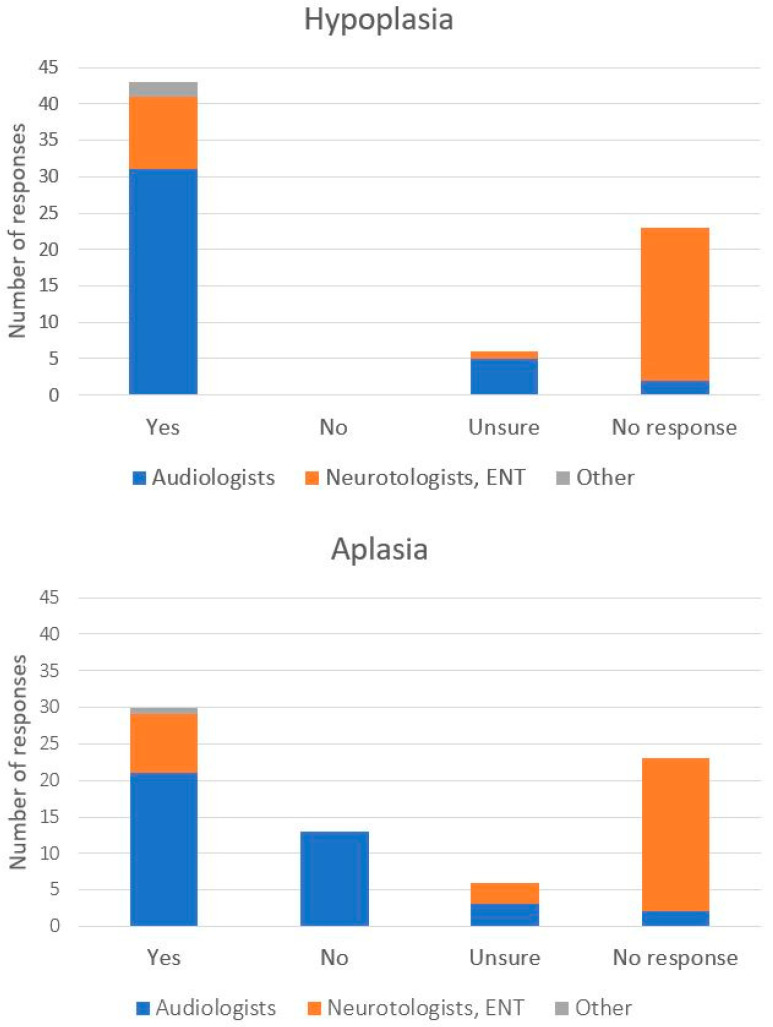
Number of participant responses to the question (s) “Do you think there are instances where cochlear implantation is appropriate for a pediatric patient with 8th nerve _______?” with the blank space before the question mark referring to hypoplasia (**top**) or aplasia (**bottom**). Distribution of professional roles of participating health care professionals is marked by color within each bar.

The majority of participants (60.2%) agreed that there are instances when CI is an appropriate treatment option for a pediatric patient with hypoplasia, while (8.3%) responded ‘Unsure’ or did not provide a response (31.9%). However, the percentage of positive responses was considerably greater among responding audiologists (81.6%) than among Neurotologists and Otolaryngologists (26.3%). For aplasia, a smaller percentage of responses favored CI (41.7%), although it was still larger than any other single category: i.e., those who marked ‘Unsure’ (8.3%), did not provide a response (31.9%), or answered ‘No’ (18.1%). Audiologists were again more likely to provide a favorable response for CI (55.3%) than neurotologists or otolaryngologists (25.0%). When responding further, if CI rather than ABI is a preferred treatment option for a pediatric patient with CN hypoplasia or aplasia, more respondents chose CI (29.2%) than ABI (9.7%), while the majority did not provide a response (45.8%) or responded neither (15.3%). The proportion of positive CI responses was also higher among audiologists (36.8%) than neurotologists and otolaryngologists (18.7%) (Figure 1).

An evaluation of individual free-text responses provided by the participants to support their choices in favor of CI over ABI provided further insight into their treatment preferences. Reasons for preferring CIs tended to be based on one or more of the following summary statements: (a) CI are associated with a lower medical risk and are less invasive, (b) CIs should be attempted first and outcomes evaluated before proceeding to ABI, (c) an expectation that CI outcomes will exceed ABI outcomes, or (d) practical concerns such as fewer places where ABI can be performed and followed up, along with more stringent qualifications for surgery. Although fewer free-text responses were provided by those who did not indicate a preference for CI over ABI, their stated reasons also included an expectation of better outcomes with ABI, especially in cases of aplasia. Notably, many responses, including those who selected ‘Neither’, advocated for a nuanced case-by-case approach with an emphasis on other patient-specific medical factors and the availability of support resources.

The discrepancy in outcome expectations among clinicians who favored CI vs. those who favored ABI could have resulted from participants’ individual clinical experiences. Among those participants who themselves previously worked with patients with CNDs, 55% reported observing cases where no auditory perception was achieved. However, there were more positive CI clinical outcomes reported with the 88.3% majority (20/36) indicating that they observed sound awareness, 66.7% (24/36) observing speech understanding with need for visual cues, and 27.7% (10/36) reported observing speech understanding with no need for visual cues. Overall, these responses show that CIs are preferred more frequently over ABI, with an overarching emphasis on case-by-case decision-making (see Table 2 for sample explanations).

The need for a patient-centered approach with a focus on patient and family counselling was also apparent in free-text responses among 43.1% of participants who indicated that there are extra steps needed to determine CI candidacy for a patient with CNDs. This was notable as none of the survey questions explicitly asked about a patient-centered approach, yet 31 professionals determined it necessary to include in their reasoning. Clinicians’ responses acknowledged the often variable and unpredictable outcomes and suggested additional steps to reduce uncertainty and establish and manage CI expectations. In addition to counselling, these included additional imaging (computed tomography (CT) and MRI) and electrophysiological assessments (evoked auditory brainstem response (eABR, EABR)), a multidisciplinary approach (social work, speech-language pathology, occupational/physical therapy, performing vision screenings, and genetic testing) as well evidence of participation in early intervention, previous hearing aid use, participation in Listening and Spoken Language (LSL) and Auditory Verbal Therapy (AVT).

When asked specifically if structural anatomical factors are the best predictors of success with CI, only 22.2% of all participants responded positively, while 29.2% did not agree, and 48.6% refrained from answering. A positive response was more likely among audiologists (26.3%) than neurotologists and otolaryngologists (12.5%). Among structural factors considered in establishing CI candidacy, the majority of responses referenced cochlear malformations (43.1%), nerve diameter (36.1%), the “number of nerves” (25.0%), and nerve fibers (15.2%) in the internal auditory meatus (IAM). A further examination of free-text responses explaining these choices revealed that most, including those who responded ‘yes’, qualified their answer to indicate that structural factors alone are not sufficient predictors.

When asked about the type of imaging utilized when considering CI candidacy in this population, Magnetic Resonance Imaging (MRI) was chosen most often by 50% of all respondents, followed by Computed Tomography (CT) (38.9%), 3D Constructive Interference in Steady State (CISS) (9.7%) and 3D Fast Imaging Employing Steady State (FIESTA) (11.1%). Most of those responding chose more than one imaging method. Specifically, all but one participant who selected CT scan also selected an MRI, while all participants who selected 3D CISS or 3D FIESTA also selected MRI, CT scan, or both.

Notably, several participants commented that as audiologists, they are not involved in the evaluation of structural factors during CI candidacy determination. Others also commented on limitations inherent in imaging studies, suggesting that imaging cannot definitively determine nerve presence or function. Some clinicians observed that at times, patients with seemingly poor structural indicators have achieved better outcomes than those with better structural profiles. Given such variability, free-text responses tended to favor a multifactorial approach to patient success that includes ensuring family commitment and an established educational support system, evaluation of patients’ comorbidities and cognitive status, as well as sufficient access to qualified clinical CI services.

## 4. Discussion

A key question when considering treatment options for a pediatric patient with CND is whether a CI recommendation is warranted or other approaches, including ABI, should be pursued instead. Although traditionally CND has been viewed as a contraindication for CI [4], several recent reports also indicated CI benefit in this population [4,8,9]. Given mixed findings and a lack of clear guidelines, the present study examined current clinical perspectives on CI in cases of hypoplastic and/or aplastic CN by conducting a survey among US hearing health care professionals. While the sample size was modest and a formal a priori power analysis was not conducted, this study was designed as an exploratory survey to capture a range of clinical perspectives regarding CI in the context of CND. Overall, our findings indicate a general lack of consensus and reflect a variety of divergent clinical perspectives. While most clinicians, and especially audiologists, indicated support for CI in cases of hypoplastic CN, fewer favored CI in cases of aplasia. However, many believe that CI should be considered prior to ABI as a preferred treatment option, unless there are strong indications that the patient will not obtain any CI benefit.

Reasons provided in support of specific treatment choices included imaging limitations, a lack of strong evidence base, and individual experiences with this population. Further, some participants stated that ABI carries higher medical risks than CI and is associated with poorer outcomes, and therefore, the potential for CI benefit should be carefully ruled out before proceeding with ABI. Importantly, free-text responses overwhelmingly advocated a patient-centered case-specific approach to accommodate a multifactorial nature underlying patient success through counselling, realistic expectations, multidisciplinary team evaluation, family commitment, and access to necessary medical care. A variety of participants’ perspectives is illustrated by sample responses by those in favor of CI, ABI, or neither, as shown in Table 2.

Encouragingly, there is a growing body of research on implantation with CND that has the potential to further inform clinical care for this population. While the present study was being conducted, three recent systematic reviews, including two meta-analyses, of implantation in cases of hypoplasia and aplasia were published [4,8,9]. All three reviews noted better outcomes for hypoplastic CN cases, with more variable outcomes for aplasia. Furthermore, all three reviews emphasized that CND should no longer be a contraindication for CI, while also acknowledging numerous limitations in the current evidence base. These limitations include small sample sizes in published reports [4,8,9], low quality of evidence [8], inconsistencies in imaging protocols, interpretation, and incomplete visualization [4,9], variability in outcome measurements and subjectivity [4,9], variability in follow-up times [4], and potential bias toward positive outcomes [4].

The conclusions of the three review studies are generally consistent with perspectives expressed by clinicians in our survey. The majority of collected responses favored CI over ABI unless it could be clearly established that CI would not provide benefit. This perspective is also reflected in the current otological literature, which states that ABI is indicated when CI does not provide adequate benefit [3]. Alahmadi et al. [8], however, indicated that ABI has the potential for greater outcomes than CI in CND, with higher CAP, Speech Intelligibility Rating (SIR), and behavioral response and speech perception scores. It should be noted that Alahmadi et al.’s [8] review included only four studies addressing ABI, all of which had populations of 12 or fewer patients. This highlights the paucity of research reporting on ABI in this population and indicates the need for further study. Furthermore, since the best outcomes for ABI in pediatric populations occur before the age of two [6], depending on the particular patient, concurrent implantation of CI and ABI may be considered [3]. As research on implantation with CND continues to accumulate, it will provide a stronger evidence base for developing CI and ABI guidelines and patient-centered protocols.

Another significant factor contributing to the uncertainty in clinical decision making about CI in this population is imaging. Presently, results of imaging studies can be associated with considerable uncertainty due to incomplete visualization [3,4,7,10,14,19], a lack of consistent protocols during imaging and in interpretation [9,14], and disagreement about what constitutes hypoplasia [3,9]. Nevertheless, as a decisive criterion for CND diagnosis, imaging plays an important role in determining candidacy for CI in cases of CND. Our results confirm that most clinicians are aware of imaging limitations, and only a small number of participants consider structural factors the best predictors of CI success. The majority overwhelmingly emphasize other factors that need to be considered, including the patient’s medical history, comorbidities, family, and institutional support systems. The discrepancy between structural assessment provided by imaging and patient success was also highlighted in several free-text responses, which explicitly acknowledged that better outcomes were sometimes observed in patients with seemingly poorer CN preservation as revealed by imaging studies. Future advances in imaging and electrophysiologic assessment methods, combined with more consistent use of settings and parameters across clinics, may increase the accuracy of clinical prognostication associated with the structural factors.

The present results also reveal differences in perspectives on CI in CND between audiologists and neurotologists/otolaryngologists, with audiologists generally viewing CI in CND more favorably. These differences may be related to the differences in aspects of patient care associated with these professional roles when determining CI candidacy. Neurotologists customarily determine surgical eligibility through medical history and imaging, both to assess the inner ear integrity and CN anatomy, and to identify any contraindications to surgical intervention. Audiologists evaluate candidacy through diagnostic testing to determine the presence and nature of a hearing loss via electrophysiological testing and behavioral audiometry, while also estimating whether a patient will receive sufficient benefit from hearing aids alone. When assessing long-term outcomes and care, both neurotologists and audiologists are integral members of a patient’s clinical care team, with medical management overseen by neurotologists and CI programming and auditory development monitored by audiologists. These differences in perspectives highlight the importance of a multidisciplinary approach to decision-making to determine the optimal course of treatment for each individual patient.

A considerable impediment to estimating future CI benefit for patients with CND arises from inconsistent use of outcome measures. A systematic review and meta-analysis by Chennareddy et al. [4] notes a high variability in the use of speech and auditory outcome measures, preventing the ability to do a meta-regression. Follow-up times also varied across studies, ranging from six months to five and a half years. Given many developmental changes that occur during the first years of life, this range in follow-up times is problematic for choosing appropriate outcome measures, as different abilities and norms are most informative for different age groups and can vary from auditory awareness to language milestones. Thus, evaluation of CI benefit following implantation will also depend on the age of implantation and post-CI follow-up interval, complicating the setting of age-specific post-CI outcomes and the development of consistent protocols. This challenge is further exacerbated by frequent comorbidities that accompany CND, with estimates as high as half of children with CND having an additional comorbidity that affects neural development [9]. Such high prevalence of comorbidities needs to be taken into account by any comprehensive clinical guideline for CI in this population.

In conclusion, our findings indicate that the majority of hearing health care professionals consider CI a viable treatment option for children with CND. Furthermore, most clinicians recognize significant limitations in the current objective measures of CN function and large variability inherent in this population, advocating for nuanced case-by-case decision-making rather than a one-size-fits-all approach. The current clinical practices reported in our survey are thus consistent with the current American Cochlear Implant Alliance (ACIA) CI guidelines [20] as well as with the conclusions in the most recent systematic reviews [4,8,9]. Clinicians’ responses acknowledged multiple factors influencing decisions about treatment options, such as surgical risk, accessibility of specialized centers, anatomical considerations (aplasia vs. hypoplasia), potential outcomes, and family expectations. Overall, responses tend to favor an interdisciplinary, individualized, and patient-centered approach to predicting success is necessary, encompassing rehabilitation, family, cognitive, and technical factors, which should be considered along with structural considerations. Nevertheless, a notable limitation of the current study is the potential for a respondent bias due to self-selection of respondents recruited via professional forums and networks. These participants may have had greater experience or interest in CI in cases of CND.

Currently, patients with CND present unique challenges for CI candidacy; the ability to successfully prognosticate CI outcomes may be improved by reducing major sources of uncertainty with associated known predictors of CI success [22]. This can be achieved by more consistent use of objective and subjective assessment measures and imaging protocols across clinics to further support the expansion of an evidence base. While the conclusions of the current study are limited by a relatively small participant sample, future studies with larger samples can, in turn, be used for building predictive algorithmic models for this population and lead to the development of comprehensive clinical guidelines and clinical decision aids (such as decision trees) that can further inform clinical judgments. As more evidence becomes available, a follow-up survey similar to the current study can then be performed to monitor the evolution of clinical practices and perspectives on CI for children with CND.

## Figures and Tables

**Table 1:** Participant characteristics.

Participant Characteristics	N = 72
Professional Role	
Audiologist	52.7% (38)
Neurotologist	40.3% (29)
Otolaryngologist	4.2% (3)
Other	2.8% (2)
CI Experience (Yrs)	
Less than 3	9.7% (7)
3–6	9.7% (7)
6–10	9.7% (7)
More than 10 years	70.8% (51)
Worked in Cochlear Implant Program Clinical Site	
Yes	95.8% (69)
No	2.8% (2)
No response	1.4% (1)
Worked with Pediatric Patients who have CN aplasia/hypoplasia	
Yes	50% (36)
No	12.5% (9)
No response	37.5% (27)

**Table 2:** Sample explanations provided by participating clinicians in support of their responses to the previous multiple-choice question: “Do you think a cochlear implant would be the preferred treatment option over an ABI for a patient with 8th nerve aplasia or hypoplasia?”.

Preference	Explanation Provided
CI	“Really depends if there is a miniscule nerve or truly absent”
	“ABI surgery is literally brain surgery…of course it would be the preferred treatment. Does that mean it will work? Not necessarily. The management of an ABI is not a walk in the park and if you’re not programming them, please consider investigating the collaboration efforts it requires from beginning to end.”
	“Cochlear implant if possible and medically recommended based on anatomy. If the family has appropriate expectations and is able to travel to an experienced center, ABI would be preferred for true aplasia.”
ABI	“If auditory/oral communication was the main goal and there was 8th nerve hypoplasia, an ABI would likely result in better outcomes.”
	“I would say the answer depends on the rest of the child’s case history. What is their cognition like at baseline? What is their vision?”
	“ABI in non NF II patients tend to have better outcomes”
Neither	“I think this is a clinical subset of patients who are likely better off pursuing a manual language modality”
	“Every case is different. A CI should be attempted first since it is less invasive. If there is no or limited sound awareness, an ABI is an option.”
	“I don’t think we have enough data”

## Data Availability

The data that support the findings of this study are available from the corresponding author, VS, upon reasonable request.

## Abbreviations

The following abbreviations are used in this manuscript:

CI	Cochlear implant
CN	Cochlear nerve
CND	Cochlear nerve deficiency
ABI	Auditory brainstem implant
MRI	Magnetic resonance imaging
HRCT	High-resolution computed tomography
ECAP	Evoked compound action potentials
eCAEP	elicited cortical auditory evoked potentials
PBK	Phonetically balanced kindergarten
BCNC	Bony cochlear nerve canal
VCN	Vestibulocochlear nerve
CAP	Categorical auditory performance
CT	Computed tomography
eABR/EABR	Evoked auditory brainstem response
LSL	Listening and spoken language
AVT	Auditory verbal therapy
IAM	Internal auditory meatus
CISS	Constructive interference in steady state
FIESTA	3D fast imaging employing steady state
SIR	Speech intelligibility rating
ACIA	American Cochlear Implant Alliance

## Appendix A

### Complete List of Questions Included in Survey

1. Have you ever worked at a clinical site with a cochlear implant program?
2. What is your professional role in hearing healthcare?
3. How many years have you worked with cochlear implant patients?
4. Do you think there are instances where cochlear implantation is appropriate?
5. Do you think there are instances where cochlear implantation is appropriate for a pediatric patient with 8th nerve aplasia (when the auditory nerve cannot be identified in imaging)?
6. Please describe reasoning for the previous questions.
7. Have you worked with pediatric patients who have 8th nerve aplasia/hypoplasia?
8. If you responded yes to the previous question, what outcomes have you seen?
  Response options
  (a) No auditory perception
  (b) Sound awareness
  (c) Speech understanding with need for visual cues
  (d) Speech understanding without the need for visual cues
9. When a patient with aplasia or hypoplasia is identified, are there any extra steps involved in determining cochlear implant candidacy?
10. If you responded ‘yes’ to the question above, if possible please describe any additional measures taken.
11. Select the type of imaging utilized when considering candidacy for cochlear implantation for patient 8th nerve aplasia or hypoplasia. (Select all that apply)
  Response options
  (a) Computed Tomography Scan
  (b) Magnetic Resonance Imaging
  (c) Three-Dimensional (3D) Constructive Interference in Steady State (CISS)
  (d) Fast Imaging Employing Steady-State Acquisition
  (e) Other
  (f) No imaging used
12. If ‘Other’ was selected for the question above please describe
13. Select all factors used when considering candidacy for cochlear implantation for patient 8th nerve aplasia or hypoplasia. (Select all that apply)
  Response options
  (a) Nerve diameter
  (b) Number of nerves in IAM
  (c) Number of nerve fibers
  (d) Cochlear malformations
  (e) Other
  (f) None of the above
14. If ‘Other’ was selected for the question above please describe.
15. For this population, are structural factors the best indicators of success?
16. If possible, please describe why for the previous question.
17. Do you think a cochlear implant would be the preferred treatment option over an ABI for a patient with 8th nerve aplasia or hypoplasia?
18. If able, please explain the reasoning for your answer to the question above.
19. Are there specific studies or rationale that you use as support of clinical decision-making? (If possible please cite)

## References

[1] Crowson M.G., Semenov Y.R., Tucci D.L., Niparko J.K. (2018). Quality of life and cost-effectiveness of cochlear implants: A narrative review. Audiol. Neurootol..

[2] Mudry A., Mills M. (2013). The early history of the cochlear implant: A retrospective. JAMA Otolaryngol. Head Neck Surg..

[3] Freeman S.R., Sennaroglu L., Lloyd S.K.W., Donnelly N.P. (2018). Management of cochlear nerve hypoplasia and aplasia. Advances in Hearing Rehabilitation.

[4] Chennareddy S., Liu K.H., Mavrommatis M.A., Kao D.D., Govindan A., Schwam Z.G., Cosetti M.K. (2024). Cochlear implantation in pediatric cochlear nerve deficiency: A systematic review and meta-analysis. Laryngoscope.

[5] Kumar P., Bhatarai P., Jayan A. (2021). Auditory brainstem implants in children with inner ear anomalies: An Indian perspective. Indian J. Otolaryngol. Head Neck Surg..

[6] Yousef M., Mesallam T.A., Almasaad A., Alhabib S., Hagr A., Alzhrani F. (2022). Cochlear implantation versus auditory brainstem implantation in children with auditory nerve deficiencies. Eur. Arch. Otorhinolaryngol..

[7] Peng K.A., Kuan E.C., Hagan S., Wilkinson E.P., Miller M.E. (2017). Cochlear nerve aplasia and hypoplasia: Predictors of cochlear implant success. Otolaryngol. Head Neck Surg..

[8] Alahmadi A., Abdelsamad Y., AlAmari N.A., Alyousef M.Y., Al-Momani M., Altamimi F.N., Alhabib S.F., Hagr A. (2024). Hearing implants in pediatrics with cochlear nerve deficiency: An updated systematic review. Eur. Arch. Otorhinolaryngol..

[9] Maturi J.R., Noij K.S., Babu V., Creighton F.X., Galaiya D., Jenks C.M. (2024). A systematic review and meta-analysis examining outcomes of cochlear implantation in children with bilateral cochlear nerve deficiency. Otol. Neurotol..

[10] Choe G., Kim Y.S., Oh S.H., Lee S.Y., Lee J.H. (2022). Functional outcomes of cochlear implantation in children with bilateral cochlear nerve aplasia. Medicina.

[11] Han J.J., Suh M.W., Park M.K., Koo J.W., Lee J.H., Oh S.H. (2019). A predictive model for cochlear implant outcome in children with cochlear nerve deficiency. Sci. Rep..

[12] Agarwal P., Gupta Y., Mundra R.K. (2023). Role of imaging in evaluating patients for cochlear implantation. Indian J. Otolaryngol. Head Neck Surg..

[13] Chao X., Wang R., Luo J., Wang H., Fan Z., Xu L. (2022). Value of preoperative imaging results in predicting cochlear nerve function in children diagnosed with cochlear nerve aplasia based on imaging results. Front. Neurosci..

[14] Kari E., Gillard D.M., Chuang N., Go J.L. (2022). Can imaging predict hearing outcomes in children with cochleovestibular nerve abnormalities?. Laryngoscope.

[15] Yigit O., Ertugay C.K., Yasak A.G., Server E.A. (2019). Which imaging modality in cochlear implant candidates?. Eur. Arch. Otorhinolaryngol..

[16] Govaerts P.J., Casselman J., Daemers K., De Beukelaer C., Yperman M., De Ceulaer G. (2003). Cochlear implants in aplasia and hypoplasia of the cochleovestibular nerve. Otol. Neurotol..

[17] Birman C.S., Powell H.R.F., Gibson W.P.R., Elliott E.J. (2016). Cochlear implant outcomes in cochlea nerve aplasia and hypoplasia. Otol. Neurotol..

[18] Young N.M., Kim F.M., Ryan M.E., Tournis E., Yaras S. (2012). Pediatric cochlear implantation of children with eighth nerve deficiency. Int. J. Pediatr. Otorhinolaryngol..

[19] He S., Grose J., Hang A.X., Buchman C.A. (2013). Cochlear implant-evoked cortical activation in children with cochlear nerve deficiency. Otol. Neurotol..

[20] Warner-Czyz A.D., Roland J.T., Thomas D., Uhler K., Zombek L. (2022). American cochlear implant alliance task force guidelines for determining cochlear implant candidacy in children. Ear Hear..

[21] Harris P.A., Taylor R., Minor B.L., Elliott V., Fernandex M., O’Neal L., McLeod L., Delacqua G., Delacqua F., Kirby J. (2019). The REDCap consortium: Building an international community of software platform partners. J. Biomed. Inform..

[22] Shafiro V., Harris M.S., Ramirez B., Du L., Moberly C. (2025). Accuracy and variability in clinical predictions of speech recognition outcomes for cochlear implant users. Int. J. Audiol..

